# Phosphite treatment can improve root biomass and nutrition use efficiency in wheat

**DOI:** 10.3389/fpls.2022.1017048

**Published:** 2022-10-31

**Authors:** Umar Mohammed, Jayne Davis, Steve Rossall, Kamal Swarup, Nathan Czyzewicz, Rahul Bhosale, John Foulkes, Erik H. Murchie, Ranjan Swarup

**Affiliations:** ^1^ Division of Plant and Crop Science, School of Biosciences, University of Nottingham, Nottingham, United Kingdom; ^2^ Mars Petcare, Melton Mowbray, United Kingdom; ^3^ Future Food Beacon of Excellence, University of Nottingham, Nottingham, United Kingdom; ^4^ Centre for Plant Integrative Biology, University of Nottingham, Nottingham, United Kingdom

**Keywords:** phosphite, biostimulants, wheat, oilseed rape, resource use efficiency, nutrition use efficiency, nitrate reductase

## Abstract

Phosphite represents a reduced form of phosphate that belongs to a class of crop growth-promoting chemicals termed biostimulants. Previous research has shown that phosphite application can enhance root growth, but its underlying mechanism, especially during environmental stresses, remains elusive. To uncover this, we undertook a series of morphological and physiological analyses under nutrient, water and heat stresses following a foliar application in wheat. Non-invasive 3D imaging of root system architecture directly in soil using X-ray Computed Tomography revealed that phosphite treatment improves root architectural traits and increased root biomass. Biochemical and physiological assays identified that phosphite treatment significantly increases Nitrate Reductase (NR) activity, leaf photosynthesis and stomatal conductance, suggesting improved Nitrogen and Carbon assimilation, respectively. These differences were more pronounced under heat or drought treatment (photosynthesis and photosystem II stability) and nutrient deficiency (root traits and NR). Overall our results suggest that phosphite treatment improves the ability of plants to tolerate abiotic stresses through improved Nitrogen and Carbon assimilation, combined with improved root growth which may improve biomass and yield.

## Introduction

Global food security is a key challenge facing world agriculture (IAASTD report, 2009; Royal Society, 2009; Godfray et al., 2010; Wheeler and von Braun, 2013; Myers et al., 2017). The world population is estimated to reach ~8.3 Bn by 2030 with the majority of that increase occurring in the developing world (IAASTD report, 2009; Royal Society, 2009). The need to feed this growing population sustainably and against the significant threats to food crop harvests arising from climate change could not be more pressing (Hirabayashi et al., 2013; Ray et al., 2013; Ray et al., 2015). With no more agricultural land available, increases in food production of between 40-50% must be achieved through a sustainable intensification of agriculture to meet the future food demand (Wheeler and von Braun, 2013). This includes both improving and developing more diverse agricultural systems for low input agriculture against a backdrop of increasing climate unpredictability that can severely affect crop production (Wheeler and von Braun, 2013). There have been several studies and reports that suggest that improvement in root architecture can have profound impact in improving crop productivity and resource use efficiency (Lynch, 2011; Lynch, 2013; Lynch et al., 2014; Lynch, 2015; Lagunas et al., 2019; York et al., 2013)

Biostimulants are a class of biologically active chemicals that are added to plants during growth or to seeds before sowing that have been shown to have beneficial effects on growth and development (Brown and Saa, 2015; Yakhin et al., 2017; Xu and Geelen, 2018). Widely used biostimulants are typically derived from seaweed extracts and both crude extracts and more refined components rich in amino acids and peptides and polysaccharides are used. Seaweed based biostimulants have been shown to improve plant tolerance to abiotic stresses and have also been implicated in improving nutrient uptake (Khan et al., 2009; Conrath, 2011; Nair et al., 2012; Savvides et al., 2016; Rouphael and Colla, 2020). Similarly, protein hydrolysate based biostimulants have been reported to improve germination rate, plant growth and productivity in a range of crops (Colla et al., 2017). The biostimulant market is a multi-billion-dollar industry (Sible et al., 2021): based on Market date forecast, Market and Markets and Dunham Trimmer European Biostimulants Industry Council (EBIC) estimate biostimulants market in EU alone to be in the range of 1.5- 2 billion USD in 2022 with a compound annual growth rate (CAGR) of 10-12% emphasizing its major importance in agriculture (EBIC, 2022).

Biostimulants are not nutrients, nor fertilizers, or pesticides and can affect plant growth and development in a variety of ways throughout the life cycle of the crop, from seed germination to plant maturity (Yakhin et al., 2017; Fleming et al., 2019). They have been shown to improve crop quality, yield and tolerance to abiotic influences (Conrath, 2011; Savvides et al., 2016) and therefore can become a key component in integrated sustainable crop production playing an important role in securing yields and increasing efficiency (Brown and Saa, 2015; Yakhin et al., 2017; Xu and Geelen, 2018). If we understood how they function we may be able to develop new traits and mechanisms to enhance yield, however this information is lacking.

Here, we focused our attention on phosphite based biostimulants. Phosphite (Phi) is a reduced form of phosphate (Pi) and has been shown to have growth promoting properties in nutrient-replete conditions in oranges, celery, satsuma, wheat, oilseed rape and several other plants (Albrigo, 1999; Lovatt and Mikkelsen, 2006; Thao and Yamakawa, 2009; Rossall et al., 2016) but not in all cases (Carswell et al., 1996; Carswell et al., 1997; Forster et al., 1998; Ticconi et al., 2001; Varadarajan et al., 2002; Thao and Yamakawa, 2009). Phosphites cannot be used as a sole source of phosphorous as phosphites cannot replace phosphate in biological reactions (Thao and Yamakawa, 2009). There is no evidence that phosphite can replace phosphate whereas phosphite to phosphate conversion by the soil micro bacteria is too slow to provide any growth benefits (Thao and Yamakawa, 2009).

The mode of action of phosphite is still unclear, however, there is some evidence that it is root-specific, and not related to any role as pesticide or fertilizer. In a glasshouse study, carried out in axenic culture, using wheat, oilseed rape, sugar beet and ryegrass Rossall et al. (2016) showed that phosphites improved root biomass by about 30% and seemed to be acting as a specific biostimulant of root growth. Roots provide the means of capturing nutrients and water required to generate a productive photosynthetic canopy. Root anatomical properties such as root length, density, depth, root front velocity and angle could also be utilized to improve resilience in resource-poor conditions to enhance capture of water and mineral elements (Ahmadi et al., 2014). Therefore, phosphites provide scope to increase arable resilience and sustainability by potentially reducing the dependence on expensive nutrient inputs (Panda et al., 2012).

Bread wheat (*Triticum aestivum* L.) provides 20% of the calorific and protein intake in the human diet (Shiferaw et al., 2013). In the UK, grain yields typically reach between 40-95% of their yield potentials (Gobbett et al., 2017) causing large yield-gaps. Here we provide a substantial understanding of physiological mechanisms by which phosphite improves root growth in the UK winter wheat. We also show that improvement of root traits correlates with improvement in both nitrogen (N) and shoot carbon assimilation. We show that phosphite treatment increases the activity of Nitrate Reductase, which is a key enzyme in N assimilation. We propose that phosphite treatment improves plant tolerance of abiotic stresses through improved N and C assimilation which is in turn associated with improved root growth.

## Materials and methods

### Plant materials and growing conditions

KWS Siskin was used for most experiments including nutrient strength, water and heat temperature and X-ray micro CT imaging experiments to its versatility allowing it to grow in a diversity of sites in the UK. It is a high yield cultivar with a disease resistance rating of 6.5 for *Septoria trictici* and a high rating for mildew and yellow rust resistance on the UK recommended list (AHDB, 2019). In addition, seven elite UK winter wheat varieties; Evolution, Diego, Leeds, Revolution, Solstice, Siskin and Skyfall were used for initial 2D root phenotyping studies.

Seeds were sieved through a calibrated graduated sieve (Scientific Laboratory Supplies Ltd. Hessle, UK) and a fraction of 2.8-3.35 mm was selected for experiments. Selected seeds were surfaced sterilized using 75% ethanol (1 min) and 30% sodium hypochlorite (10 min) and washed thoroughly using sterile water.

### Germination paper assays

Sterilized seeds were germinated on a moist filter paper (Whatman) for 5-7 days at 4°C and 2 days in a controlled environment room set at 16/8h photoperiod (20/15°C). The uniformly germinated seeds (~0.5 cm radicle root) were transferred on to the blue paper germination pouches soaked in 1/6^th^ strength Hoagland’s 2.0 (Sigma) (Hoagland and Arnon, 1950) hydroponic media (pH 6.0).

4-5 days old seedlings were treated with a potassium phosphite based formulation (1 L ha-1 + 0.1% NA 13 wetting agent) as a foliar spray, using a calibrated, hand-held spray gun in a volume of water equivalent to 200 L ha^-^1. Control plants were sprayed with 0.1% NA13 wetting agent only. The root impression underneath the black sheet revealed the position of each seminal root and was marked using a blue permanent marker to indicate the position of the root at the time of the foliar application.

A randomized block design was used with a minimum of 24 replicates per treatment. After 9 days (two-leaf stage), another mark was made on the new position of the roots as well as marking the newly developed roots based on the impression. The pouches were imaged on both the black sheet and the blue paper using a Nikon D600 DSLR camera as described by Atkinson et al. (2015).

Root images were processed using RootNav software as described in Pound et al. (2013) and ImageJ software (NeuronJ) for analyzing the changes in the root development after phosphite application.

### X-ray micro CT imaging experiment

Seeds were surfaced sterilized (above) and stratified at 4°C in a square petri-dish containing moist filter paper for 3 days with each seed facing down. After 3 days, they were moved to a 22°C growth chamber and the petri-dish was wrapped in aluminum foil and placed at a 65° angle to allow the root radicle to grow towards gravity.

Soil (sandy loam) was collected from a field under winter wheat at the University of Nottingham experimental farm, Sutton Bonington campus. The soil was air-dried and sieved to <2 mm before being uniformly packed into columns (7.5 cm diameter and 17 cm height). A small yellow pipette tip was placed in the middle top of the soil to 0.5 cm depth before the soil was soaked. This prevents compaction during seed transplanting. The soil moisture was saturated at field capacity 24 hr before the wheat was transplanted. Once the root radicle emerged and was 0.2 cm long, the yellow tip in the middle of the column was removed and the germinating seed was replaced in the soil with roots directly facing downward. Soil of ~1 cm thick was placed over the top to bury the seeds. A completely randomized design was used with a minimum of 10 replicates per treatment. At 4 days after transplanting, the emerging leaves were treated with a potassium phosphite based formulation (1 L ha^-1^ + 0.1% NA 13 wetting agent) as a foliar spray, using a calibrated, hand-held spray gun in a volume of water equivalent to 200 L ha^-1^. Control plants were sprayed with 0.1% NA13 wetting agent only. Ten biological replicates were made each and the columns were X-ray scanned at day 0, 6 and 12 after phosphite treatment using a Phoenix Nanotom (GE Measurement and Control Solutions, Wunstorf, Germany) micro CT scanner.

Image processing and analysis of the root system were segmented from the grey-scale micro CT images using the Volume Graphic Studio Max (VG StudioMax) software described by Tracy et al. (2013). The root volume and surface area were all extracted from the VG StudioMax while the root branching and root count were analyzed using semi-automated ROOTh imaging software.

### Growth and phosphite application experiments in controlled conditions

For all phosphite treatments hereon, a potassium phosphite based formulation 0-28-19 (O-Phyte) (Omex Ltd.) containing 31.15% PO_3_ was used. The 0-28-19 was diluted with water (5mL/L and mixed with 0.1% solution of the wetting agent NA13 (Omex Ltd). This solution containing 0.15% PO_3_ was used as foliar spray at the rate of 1L per m^2^. Control plants were sprayed with 0.1% NA13 wetting agent only.

The sterilized seeds were pre-germinated on a moist filter paper in Petri-dishes in the dark at 21°C for 3 days. Uniform-sized seeds with a root radicle of ~0.5 cm were sown in germinating plugs containing compost (John Inness, Norwich UK) and were grown in a glasshouse for 7-10 days until GS12 (Zadoks et al., 1974). Plants were then vernalized at 6°C for 4-5 weeks with a 16/8h photoperiod. Plants were then transferred into a glasshouse with 21°C/15°C day and night temperature and 16/8 h photoperiod for 7 days.

At 7 days post vernalization, plants were transplanted into deep, plastic pots filled with ~0.6-10 mm washed expanded clay pellets (Hydroleca) and maintained in this semi-hydroponic system by daily irrigation as described previously (Rossall et al., 2016). After a week in this semi-hydroponic system, phosphite and control treatments were applied as mentioned above. Root and shoots were harvested at different time points after treatment and dried at 70^0^C for three days before dry weight calculations.

### Nutrient strength experiment

Three different hydroponic media were used in the semi-hydroponic system described above. Two were commercially available media from Omex and Hortimix with a composition of NPK: 20-8-20 + Mg and S with trace elements and NPK: 15-7-30 with trace elements respectively. While the third hydroponic media was a modified laboratory formulated Hoagland’s solution with EDTA as the iron chelator. The composition (g/L) of nitrate and phosphate was reduced by half; (NH_4_)_3_PO_4_, 0.058g; Ca(NO_3_)_2_, 0.66g; MgSO_4_, 1.01g; KNO_3_, 0.304g; H_3_BO_3_, 0.114g; Cu_2_SO_4_, 0.3g; MnCl_2_(H_2_O)_4_, 0.04g; MoO_3_, 0.0008g; ZnSO_4_, 0.0092g; FeHEDTA, 0.11g. The solution was adjusted to pH 6.0.

For the nutrient strength response experiment, the required amount of the two commercial hydroponic media was diluted with water to make up either ¾ or ½ or ¼ strength.

### Mild drought experiment

At 7 days post vernalisation, plants were transplanted into a 1 m PVC constructed column containing ~0.6 – 10 mm hydroleca and irrigated with the full complement of nutrients using automated irrigation drips. 10 days after transplanting into the 1 m column, the plants were treated with a potassium phosphite based formulation as a foliar spray. The plants were set to a randomized complete block design. A week after the phosphite treatment, the irrigation was restricted and as the hydroleca is inert and does not hold water, the automated irrigator supplied 75 ml of water every 6 h to achieve a moderate drought. The plants were allowed to grow in this condition until they reached Growth Stage 65-69 i.e., mid to late anthesis before the plants were harvested for the shoot and root sampling.

### Heat stress experiment

At 7 days post vernalisation, the wheat seedlings were transferred into a pot containing a mixture of soil and compost (John Innes No.3). All other environmental conditions are the same as above with the exception of the heat treatment. After 14 days at 21°C, they were sprayed with phosphite and were immediately subjected to heat stress of 35°C for 30 days in a controlled environment chamber (Conviron, Winnpeg) followed by an increased temperature of 38°C for 5 days before reducing the temperature back to 22°C for the remainder of the experiment.

### Leaf gas exchange measurements and chlorophyll fluorescence

All leaf gas exchange measurements were taken on the uppermost, fully expanded leaf using an infra-red gas analyser (IRGA), Licor 6400XT or 6800 (Licor inc, Illinois, Nebraska). The same IRGA model was used within each experiment. Three types of measurements were made: single time point measurements of photosynthesis at light-saturation (*A*_max_), Photosynthesis versus intercellular CO_2_ concentration (*C*i, A/*C*i) analysis and light response curves as described previously by Webster et al. (2016). The photosynthetic model of Farquhar et al. (1980) was fitted using the bilinear method of the ‘fitacis’ function from the R package PLANTECOPHYS (Duursma, 2015). Except where noted, measurements were made under light-saturated conditions (1500 µmol m^-2^ s ^-1^). The block temperature was maintained at 22°C, the flow rate 500 ml min^-1^ and 60-65% humidity. The CO_2_ concentration used was 400 µmol mol^-1^ (except where noted). All measurements took between 2 and 3 minutes to achieve stability. The leaf water instantaneous water use efficiency (IWUE) and intrinsic water use efficiency values (iWUE) were obtained from the ratio of photosynthetic CO_2_ assimilation (*A*) to stomatal conductance (*g_s_*), and *A_n_* to leaf transpiration (*E*) respectively.

### Chlorophyll fluorescence

From 5 days after phosphite application, between 11:00 am and 12:00 pm; the top and fully developed leaf was wrapped with a ~3 cm x 3 cm aluminium foil around the upper middle part of the leaf. The leaf was left to dark adapt for 30 mins and the light of the growth chamber was turned off before the measurement started. Handheld FluorPen FP 100 (Photon Systems Instruments, Brno) was used to measure the maximum quantum yield (*F*_v_/*F*_m_) of the dark-adapted leaf from ten biological replicates.

For disruptive analysis, the *F*_v_/*F*_m_ was measured from excised leaf segment of 10 biological replicates of phosphite treated and untreated under two contrasting temperatures (Ferguson et al, 2020). The leaf segments were placed on a damp filter paper and the paper was encased between glass plates. The plates were placed inside a closed 800C FluorCam chlorophyll fluorescence imager (Photon Systems Instrument, Brno) to dark adapt for 1 h before the standard *F*_v_/*F*_m_ protocol was run as described by Mcausland et al. (2019).

### Spectroradiometer

Hyperspectral reflectance was measured between 11:00 am and 2:00 pm using the ASD Field Spec 4 (ASD Field Spec ^®^ 4, Boulder, CO, USA) with a spectral range from 350 – 2500 nm. The reflectance measurement was made using the leaf clip in two different leaves of the top fully expanded leaf as described by Robles-Zazueta et al. (2021). The measurements were taken in ten biological replicates. Chlorophyll content was measured on the two top fully expanded leaves using the SPAD meter (SPAD-502 meter, Konika Minolta, Japan).

### Nitrate reductase assay

Nitrate reductase assay was done as described previously (Kim and Seo, 2018). A root or shoot tissue (500 mg) was ground using liquid nitrogen and suspended in 750µl of chilled extraction buffer (250 mM Tris-HCl, pH8, 1 mM EDTA, 1 µM Na_2_MoO_4_, 5 µM FAD, 3 mM DTT, 1% BSA, 12 mM 2-mercaptoethanol, 250 µM PMSF) and centrifuged at 17,000 g for 5 min. 150 µl of supernatant was added to 850 µl of reaction buffer (40 mM NaNO_3_, 80 mM Na_2_HPO_4_, 20 mM NaH_2_PO_4_, 0.2 mM NADH) and incubated for 2 h at room temperature. 200 µl of 1% sulfanilamide and 200 µl of 0.05% N-(1-naphthyl) ethylenediamine were added to each reaction and incubated at room temperature for another 15 mins and absorbance measured at 540 nm. Protein contents were measured using the Bradford assay (Bradford, 1976) and NR activity was expressed as specific activity (units/mg protein).

### Statistical analysis

Statistical analysis was performed within the R software environment (R Core Team, 2014) and GraphPad Prism 9.01 for Windows (La Jolla, CA, USA) for figures. Student T-tests were used in comparisons between two samples. Analysis of variance (ANOVA, one-way and two-way) with posthoc Tukey’s multiple comparison procedure was used except where indicated otherwise. Pearson’s product-moment correlation coefficient was performed for all pairwise traits interactions using the ‘rcorr’ correlation analysis from the R package HMISC, using the ‘corrplot’ function (Mckenna et al, 2015).

## Results

### Phosphite treatment improves root growth in wheat

Initially we tested the effect of phosphite on young seedlings in six commercial winter wheat varieties Diego, Evolution, Leeds, Revolution, Siskin and Skyfall. Early seedling stage root phenotyping was done using 2D high throughput root phenotyping system (Atkinson et al., 2015). Four out of six commercial winter wheat varieties; Diego, Leeds, Siskin and Skyfall showed a significant increase (Tukey’s HSD P<0.05 and lower) in the seminal root length (**Supplementary Figure 1A**. The mean of the seminal root count in Diego was significantly (P<0.05) increased by 18% while its lateral root counts significantly (P<0.05) increased by 38% and Leeds by 58%). Interestingly, Evolution and Revelation did not respond to phosphite treatment indicating a varietal difference in their response to phosphite treatment.

**Figure 1:**
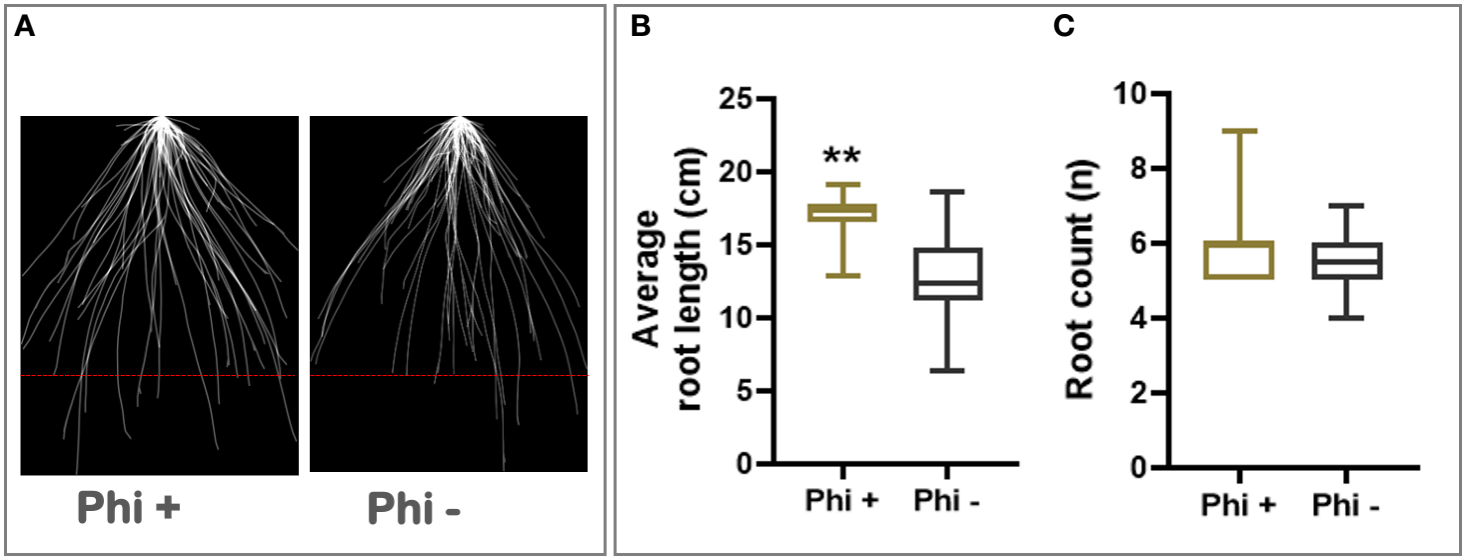
Phosphite promotes root growth in wheat seedling. **(A)** Superimposed 2D root images of young seedlings grown in low strength hydroponic media showing improvement in seminal and nodal root length at 7 days post phosphite treatment. **(B)** Seminal root length **(C)** seminal root count. All values are means ± SE (n= 25); ** indicates signifcant difference (P ≤0.01).

In addition to its response to phosphite, winter wheat cultivar Siskin is more resistant to powdery mildew infection and hence was sub-selected for further experiments under reduced nutrient strength. Using the early-stage root phenotyping set up described above, Siskin plants were grown under reduced nutrient strength with and without phosphite treatment. **Figure 1A** shows superimposed 2D images of young Siskin seedlings showing improvement in seminal and nodal root length at 7 days post phosphite treatment and reveals an increase in the length and number of the nodal and seminal roots (**Figure 1B**).

### X-ray CT imaging reveals that phosphite treatment improves root architectural traits

**Figure 2:**
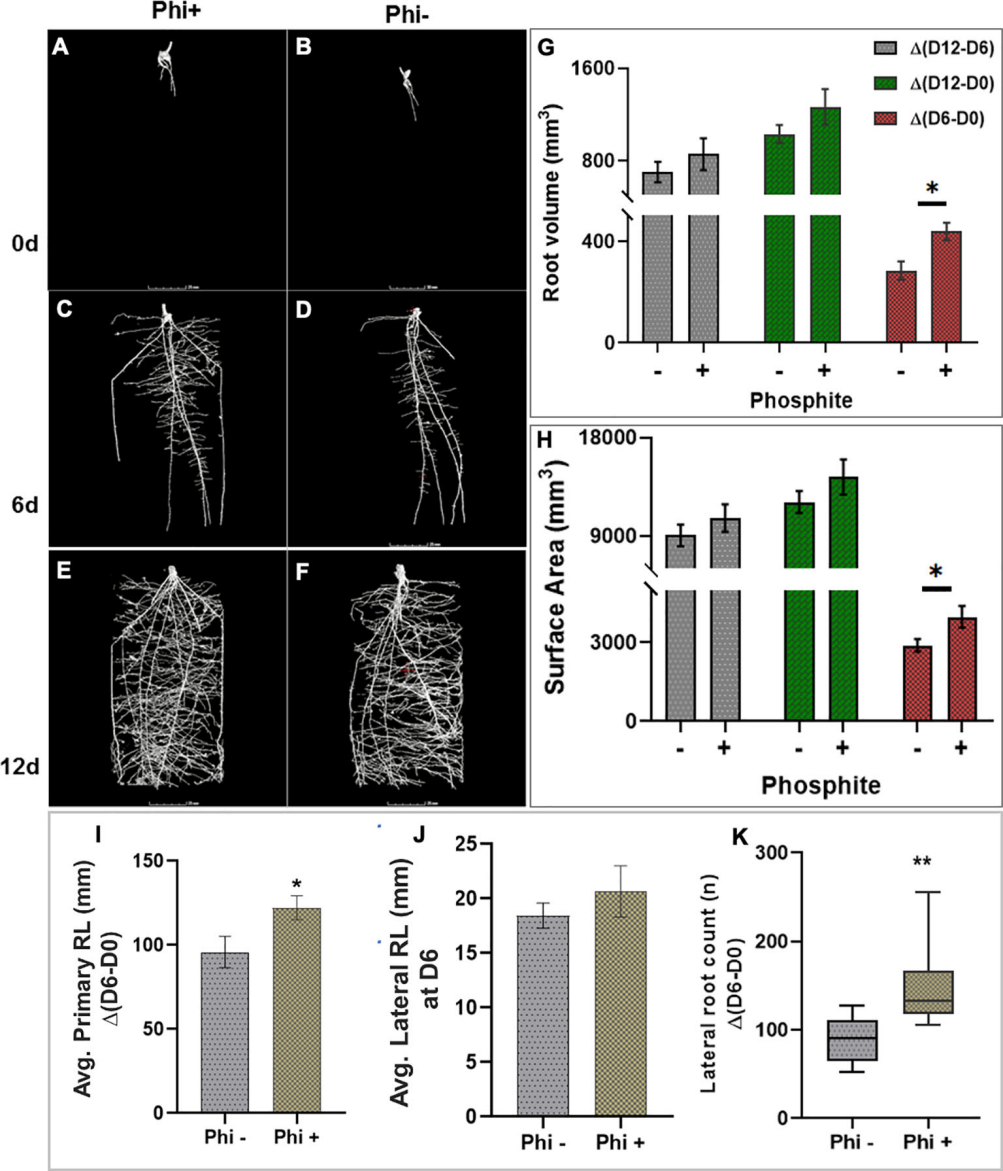
X-ray CT imaging in wheat revealed that phosphite improves root architectural traits. **(A–H)**. Time course X-ray CT imaging at 0, 6 and 12 d post treatment **(A–F)** reveals that phosphite treatment results in increase in root volume **(G)** and root surface area **(H)**. **(I–K)**. Average primary root length **(I)** average lateral root length **(J)**and lateral root count **(K)**. Wheat seedlings grown in sandy loam soil in small columns (7.5 x 17cm). 4 day old seedlings were treated with a potassium phosphite based formulation and X-RAY CT imaging performed at 0, 6 and 12 d post application. Student t test was used to compare between treatment and untreated control (*indicates significant difference *P ≤ 0.05 and **P ≤0.01). All values are means ± SE (n= 10).

To test the impact of phosphite on root growth in soil, pre-germinated wheat (variety Siskin) seeds (~1cm radicle roots) were sown into sandy loam soil in small columns (7.5 x 17cm). 4d old seedlings were treated with a potassium phosphite based formulation through the foliar spray and X-RAY CT imaging was performed at 0, 6 and 12 d post-application (**Figure 2**)

The result was computed based on the changes between time points. The differences between day 6 and day 0 of treatment are referred to as Δ(D6-D0), similarly between day 12 and 6 and between day 12 and 0 as Δ(D12-D6) and Δ(D12-D0), respectively. The CT results revealed that at 6 d post-application Δ(D6-D0) there was a significant increase in the root volume (P=0.027) and the surface area of the root (P= 0.036) by 28% and 27%, respectively. The increase in both the root volume and the surface area in response to the phosphite application can also be seen at the later time points but the increase was not significant (**Figures 2G, H**). There was also a significant increase in seminal and nodal root length (P=0.042) by 22% and lateral root count (P=0.002) by 39% (**Figures 2I, K**) 6 days after the phosphite treatment. We also found a significant positive correlation between the root biomass and lateral root count at 12 days post treatment; root surface area and root volume after 6 days post treatment (r=0.42, *r*=0.43 and *r*=0.60 respectively; **Supplementary Figure 2**). The shoot biomass also showed positive correlation with the root volume (r=0.55).

### Phosphite treatment improves root biomass under nutrient deficiency

Roots are crucial for nutrient and water uptake from the soil. To test if improved root growth can lead to improvement in nutrient uptake, the plants were grown under three different nutrient strength solutions: full strength (100%), ¾ strength (75%) or ½ strength (50%). 10-day post transfer to the hydroponic set up, plants were subjected to foliar phosphite application. The growth was monitored through detailed physiological measurements and destructive root and shoot biomass analysis (30- and 45-days post treatment).

**Figure 3:**
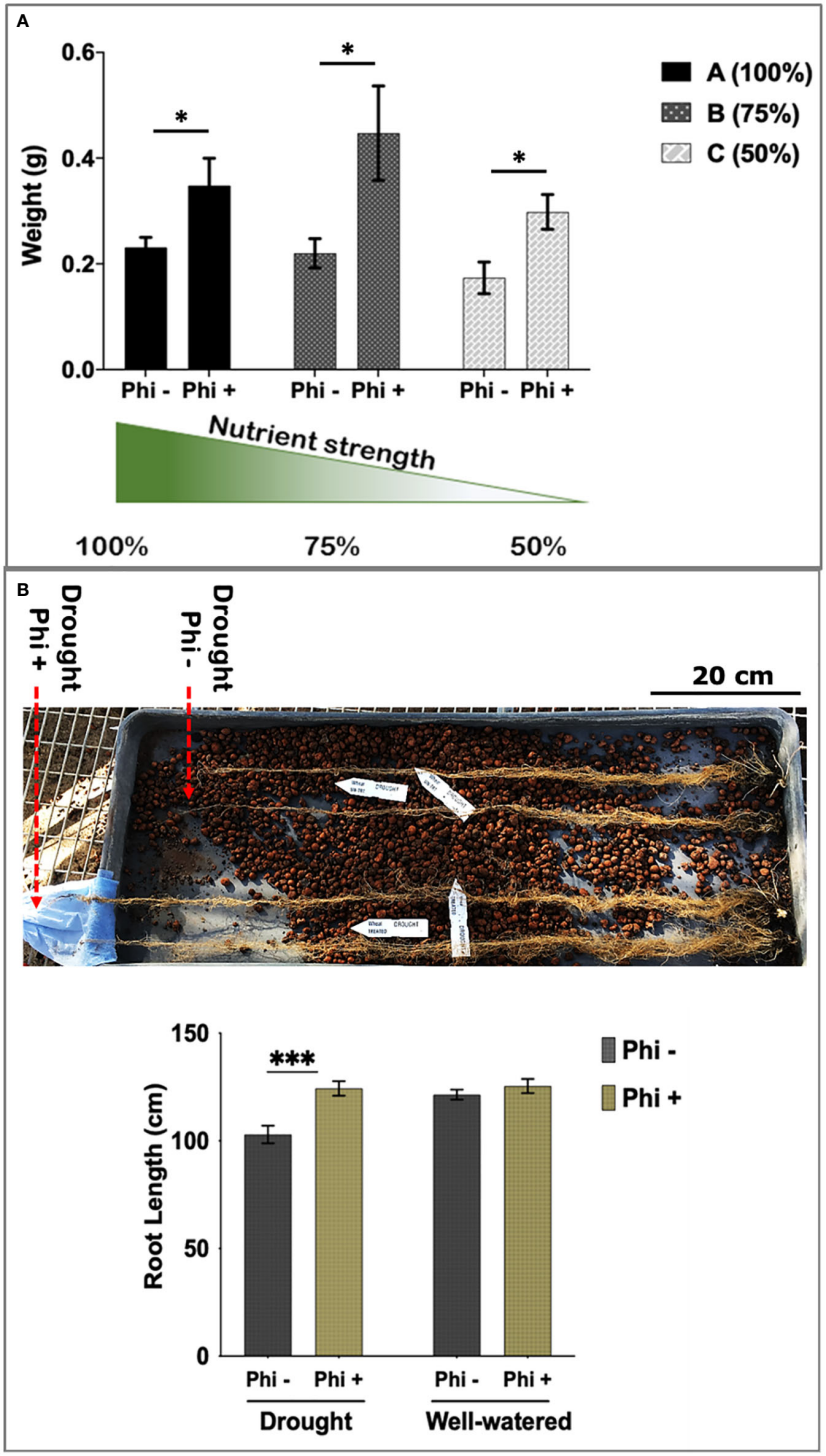
Phosphite treated plants show improved root development. **(A)** Root dry weight of Phosphite treated plants in pots under different nutrient strength of a commercial soluble fertilizer (NPK: 20-8-20) compared to untreated plants. *indicates significant difference (student’s t test P ≤ 0.05). **(B)** Deep columns were used to show an increase in maximum root length under mild drought compared to untreated plants. Arrowheads indicate end of root tips. Growth assessment 10-45 days after phosphite treatment. All values are means ± SE (n= 10-15); *indicates significant difference (*P ≤ 0.05 and ***P ≤0.001). Scale bar=20cm.

As shown in **Figure 3A**, phosphite treated plants showed a significant increase in root biomass at all three nutrient strengths compared to the control treatment. Phosphite treated plants at 75% nutrient strength solution showed the highest increase in root dry weight (51%) compared to control (P=0.0258); whereas there was a 34% increase in root dry weight at 100% strength nutrient solution (p=.0476); and 40% increase at 50% strength nutrient solution (p=0.0128) compared to control treatments. Moreover, phosphite treated plants grown in reduced nutrient strength solutions (75% and 50%) outperformed the full strength non-treated plants. All three nutrient concentrations also showed an increasing trend (17%, 22% and 25% respectively) in shoot biomass in phosphite treated plants compared to control treated plants.

To further validate these findings, we designed another experiment where plants were grown under formulated Hoaglands solution with half strength N and P and growth measurements were done at 2, 4 and 8 weeks post treatment. At all the three time points, phosphite treated plants had significantly longer (27, 20 and 39%, respectively) roots compared to the untreated control (**Supplementary Figure 3**). Results also show a significant increase in root dry weight by 28% (P=.045) in phosphite treated plants 2 weeks post application further confirming the observation (**Figure 2**) that phosphite treatment improves root growth and may help promote seedling establishment. In addition, a significant positive correlation was also seen in the root dry weight and maximum root length in both week 2 and 4 after treatment (r=0.6 and r=0.53, respectively).

Despite improvement in root growth and root dry weight, we did not detect any differences in the shoot dry weight between treated and untreated plants (**Supplementary Figure 3Biii**).

### Phosphite treatment improves root length under limited water condition

Roots can establish at depths much greater than the pots commonly used in controlled environments. To measure root depth in a restricted water experiment, seedlings were transferred to 1 m long columns containing expanded clay beads fitted with an automated drip irrigation system. Foliar phosphite treatment was done 5 days post transfer of the seedlings to these columns and water restriction was imposed a week later. Root growth assessment at 45 days after the treatment showed that phosphite treated plants have longer roots under restricted water regime (**Figure 3B**).

### Phosphite treatment improves carbon assimilation under mild water and nutrient stress conditions

**Figure 4:**
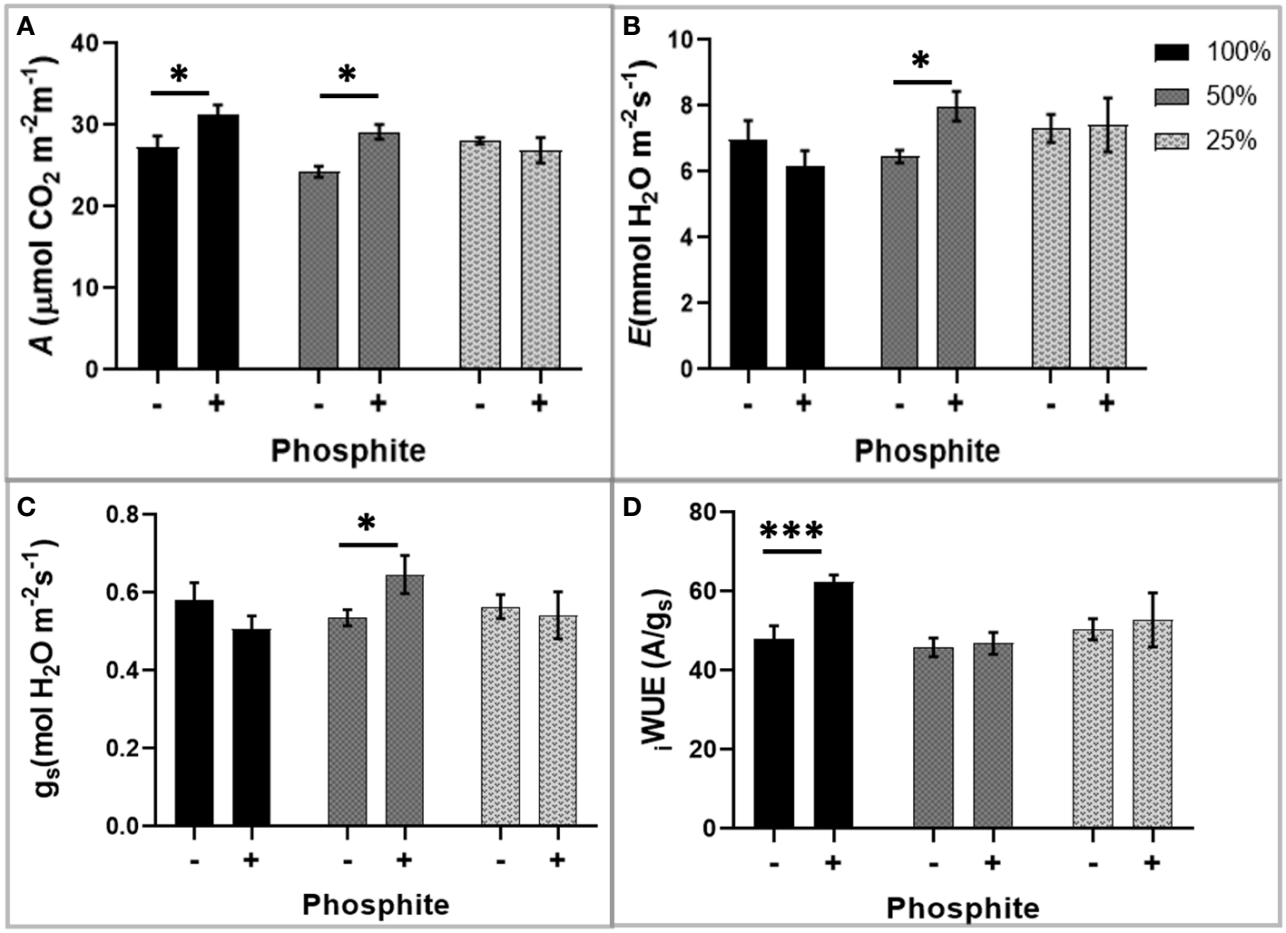
Phosphite treatment improves carbon assimilation under mild nutrient stress. Photosynthetic response of phosphite treated (+) and untreated **(-)** wheat plants grown under different nutrient strength of a commercial soluble fertilizer (NPK: 20-8-20). **(A)** Net carbon assimilation rate **(B)** Transpiration rate **(C)** Stomatal conductance **(D)** Intrinsic water use efficiency from a ratio of net photosynthesis to stomatal conductance. All values are means ± SE (n=6); *indicates significant difference (student’s t test P ≤ 0.05). ***P ≤ 0.001.

The observed alterations in root traits may have consequences for the plant capacity to supply the shoot with nutrients for synthesis of photosynthetic components and water for leaf gas exchange. To investigate this, we utilised infra-red gas analysis. We observed enhanced photosynthesis as a common trend in phosphite treated plants. At both high (100%) and moderate (50%) nutrient strength, photosynthetic capacity (*A*_max_) increased significantly by 12 and 21% respectively, after 28 days of phosphite treatment (P=0.027 and 0.001)(**Figure 4A**). Stomatal conductance (gs) and the transpiration rate at moderate nutrient strength (50%) were higher in the phosphite treated plants compared to untreated control plants (P=0.045 and 0.015 respectively; student’s t test)(**Figures 4B, C**). Intrinsic leaf water use efficiency WUE (calculated as *A*/g_s_) of phosphite treated plants in full nutrient complement also improved significantly (P=0.0004; **Figure 4D**).

Carbon assimilation and gas exchange rates were measured at 4 different time points within the first 15 days of mild water restriction (**Supplementary Figure 4**). Although not significant, the *A*_max_, the stomatal conductance (g_s_) and the transpiration rate (*E*) of phosphite treated were all numerically higher at all the 4-time points compared to non-treated under mild water stress with no alteration in WUE.

A-Ci analysis was used to separate biochemical components of photosynthesis such as the maximum rate of carboxylation by Rubisco (*V*_cmax_) and the maximum rate of electron transport for ribulose-1-5-bisphosphonate (RuBP) regeneration (*J*_max_) at 10 days of water restriction. For both the *V*_cmax_ and *J*_max_ effects were not significant comparing phosphite treatment under mild water stress to the untreated control (**Supplementary Figure 4D**).

### Phosphite treatment improves responses to heat stress

**Figure 5:**
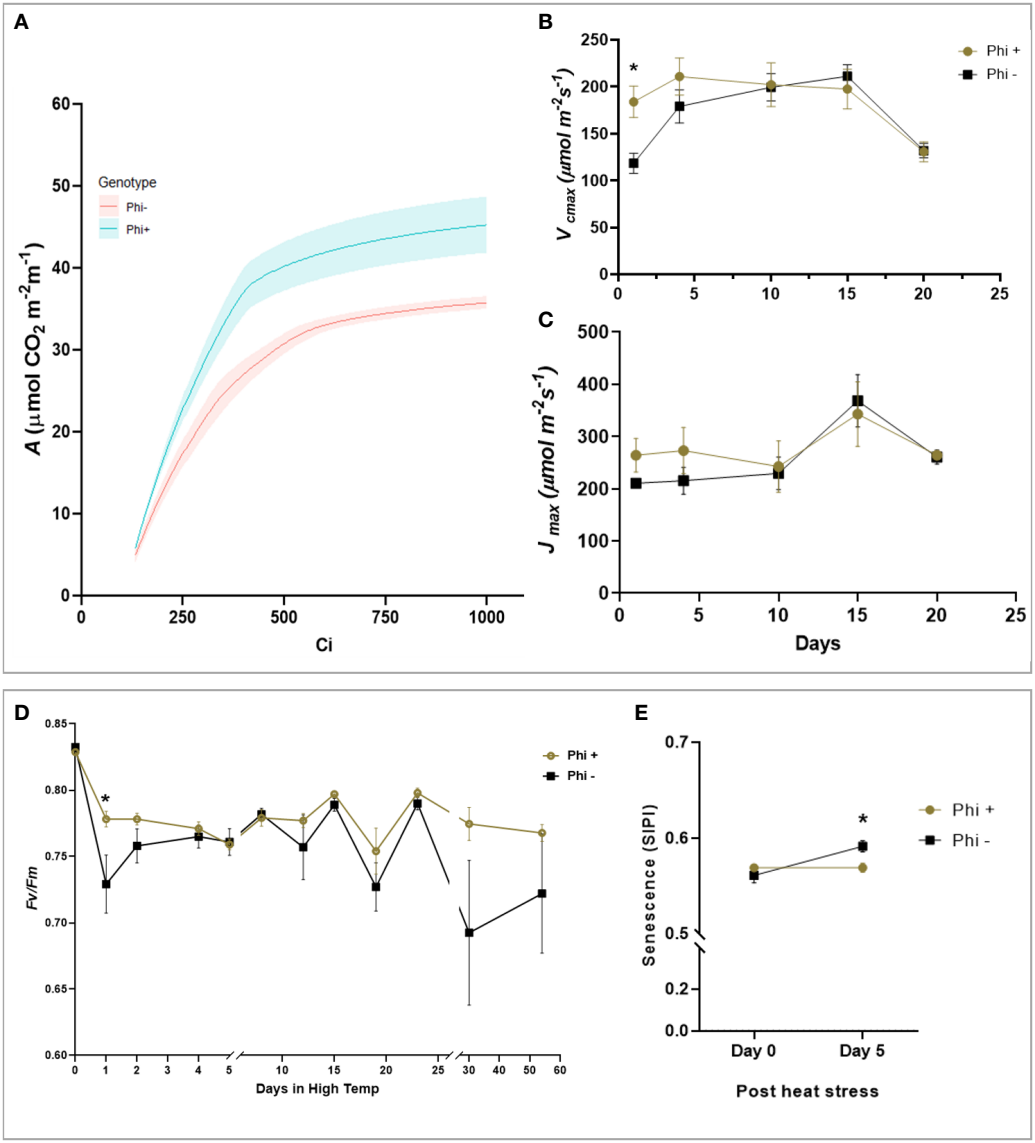
Phosphite treatment shows improve tolerance to heat treatment. **(A)**. *A* versus Ci response curve at 24hr of heat treatment. For visual clarity the lines are fitted through mean values and the shaded region show limits to the standard error of mean. **(B)**. Maximum rate of carboxylation of Rubisco (*V*_cmax_) **(C)**. Maximum rate of RuBS regeneration/electron transport (*J*_max_) **(D)**. Maximum efficiency of photosystem II after dark adaptation. **(E)** Canopy senescence (SIPI). All points are means ± SE (n= 5 - 10); (* represent significant differences between phosphite treated and untreated of the same time point (P ≤ 0.05).

During the heat stress period, the response of the maximum quantum efficiency of photosystem II (*F*_v_/*F*_m_) was measured every day in the first 5 days, followed by measurements at 5 days intervals. At 24 h after heat stress, *F*_v_/*F*_m_ of the untreated plants was significantly lower than treated (P<0.05) **Figure 5D**). When the temperature was again increased at day 30 to 38°C, there was a trend for a sharp and substantial decline in *F*_v_/*F*_m_ for the untreated plants but not treated (P=0.081). This suggests that phosphite treatment plays a role in helping to prevent photoinhibition, likely caused by damage to the PSII complex during heat stress.

To investigate this further, ACi analysis was performed during the heat stress treatment at 5-day intervals. On day 1 and 4 of the heat treatment, there was a substantial reduction in *V*_cmax_ (P=0.0169, **Figures 5A, B**) in untreated plants which mirrored the reduction in *Fv/Fm* but *J*_max_ was not significant (**Figure 5C**). Additionally, to determine if phosphite treatment has any role to play in leaf senescence under heat stress, we measured the whole plant senescence: Structural Insensitive Pigment Index (SIPI; **Figure 5E**) using the vegetative index calculated from hyperspectral reflectance at day 0 and 5 of heat stress.

### Phosphite treatment increases nitrate reductase activity

It has recently been reported that phosphite application results in an increase in nitrate reductase activity.

To test the effect of phosphite treatment on nitrate reductase, nitrate reductase enzyme activity (NRA) was measured in phosphite treated and untreated plants grown in different growth mediums (soil columns; field condition and hydroponics) and different growth conditions (varying nutrients and water levels).

**Figure 6:**
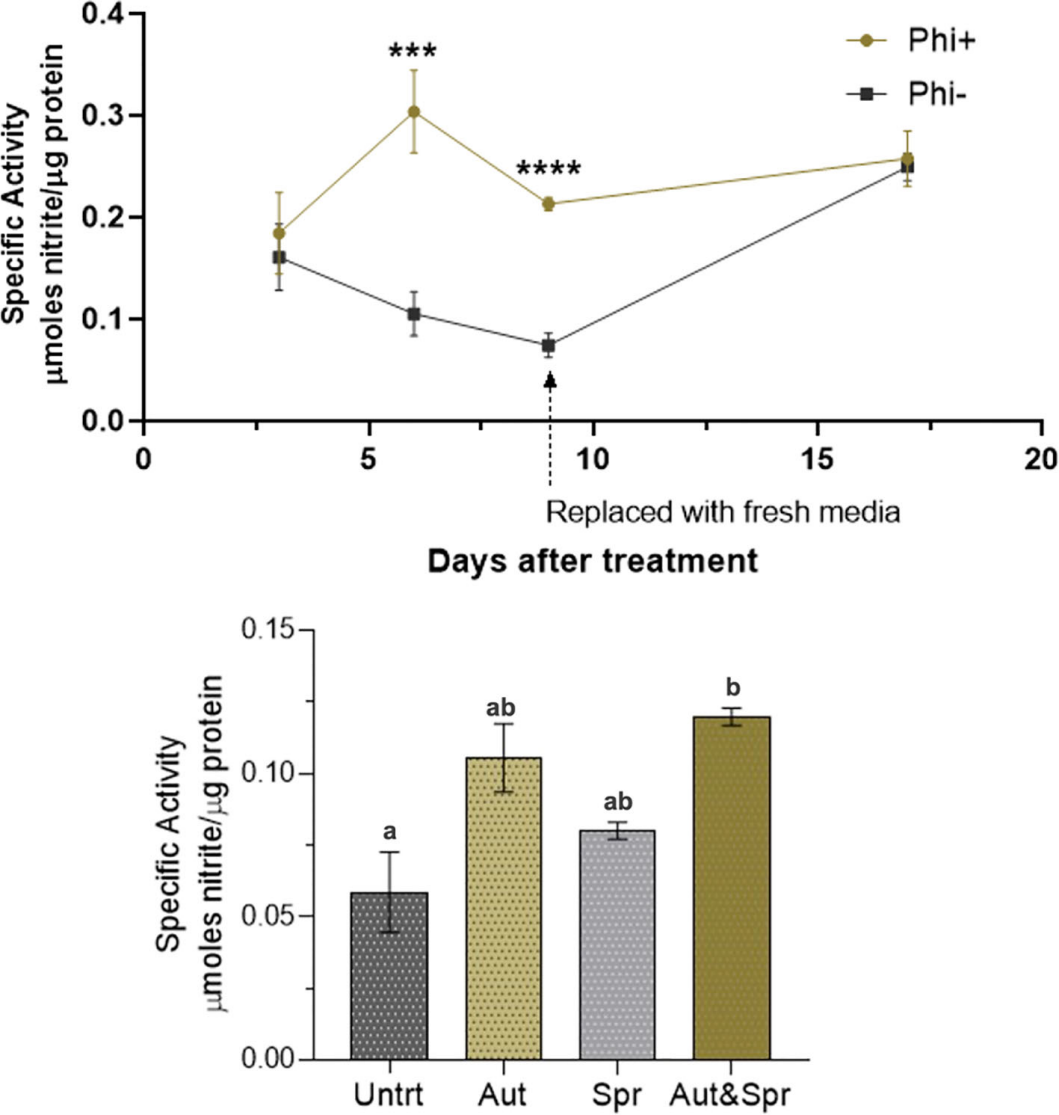
Phosphite enhances Nitrate Reductase activity. **(A)** Time-course assessment of NR activity of wheat plants grown under reduced nutrient (50% strength) from 3 days to 19 days post treatment. *indicates significant difference. (***P ≤0.001 and ****P ≤ 0.0001; student's t test) **(B)**. Wheat plants grown in the field with different times of phosphite application. Effect of phosphite on nitrate reductase activity is commonly observed under mild stress. All values are means ± SE (n= 5 - 25); Different letters denote statistical significance (P ≤ 0.05; one-way analysis of variances. All values are means ± SE (n= 5 - 25) for both **(A, B)**.

The nitrate reductase activity was measured on wheat shoots grown in ½ strength hydroponic media (**Figure 6A**). There was a significant increase in nitrate reductase activity 6 and 9d post application.

We next tested the nitrate reductase activity in field grown wheat plants. Plants were treated with phosphite in autumn, spring or both in autumn and spring. We detected a significant increase (51%) in nitrate reductase activity in dual phosphite application (autumn and spring; **Figure 6B**).

To test if the phosphite-influenced increase in nitrate reductase is only common to wheat, we measured the nitrate reductase activity of winter brassica (dicot crop) using the same cultivation methodologies. In line with wheat experiments we used ½ strength of commercial NPK 20-8-20 hydroponic media as described in the methods. Our results show that 9 days after phosphite application, there was a significant increase (44%) in the nitrate reductase activity (**Supplementary Figure 5A**). We also tested the nitrate reductase activity of seedlings growing in soil column under restricted water conditions. *Brassica napus* (var Anastasia) and *B. rapa* (var Skye) seedlings were treated with phosphite and 5 days after phosphite application, water was restricted and nitrate reductase activity was measured 12-day post treatment. Our results show both varieties (Skye and Anastasia) have a significant increase in the nitrate reductase activity by 67% and 60% respectively (**Supplementary Figure 5A**).

## Discussion

Biostimulants are emerging as a class of chemicals that promote crop growth. They also have been reported to improve plant fitness and performance under stress conditions (Ziosi et al, 2013; Brown and Saa, 2015; Bulgari et al, 2015; Bulgari et al, 2019). With increasing incidents of adverse growing conditions, the use of biostimulants to enhance crop establishment and growth is becoming more important and could be crucial for meeting future food demands.

Phosphite represents a reduced form of phosphate that has been shown to act as a biostimulant (Albrigo, 1999; Lovatt and Mikkelsen, 2006). Previous studies in wheat have shown that foliar application of phosphite enhances root growth and development in a range of plant species, typically increasing biomass by around 30% (Rossall et al., 2016) but mechanisms of action are unclear.

Here we have carried out more detailed studies on the effect of phosphite on root growth and its impact on nutrient use efficiency and above ground physiology. In early seedling stage root studies, we tested the impact of phosphite in six commercial winter wheat varieties. Four of these varieties (Diego, Leeds, Siskin and Skyfall) responded to phosphite treatment and resulted in increase in the length and number of the nodal and seminal roots (**Figure 1**). Interestingly, Evolution and Revelation did not respond to phosphite treatment indicating a varietal difference in their response to phosphite treatment. Currently it is not clear why some varieties respond to phosphite treatment and others don’t. Phosphite cannot be used as a source of P nutrition (Carswell et al., 1996; Forster et al., 1998; Schroetter et al., 2006). Phosphite can be taken up by the phosphate transporters but cannot replace Pi in most biological reactions but suppress the Pi starvation response (McDonald et al., 2001; Varadarajan et al., 2002). There is not much evidence either to suggest that phosphite can be converted to phosphate *in planta (* Thao and Yamakawa, 2009). However, there are some reports that suggest that phosphite can be converted to Pi by soil microbacteria but the conversion is very slow to account for any nutritional benefits (McDonald et al., 2001). Besides, the doses used here are very small and typically one application of phosphite is needed to promote root growth. Therefore, we rule out the possibility of phosphite as a source of P nutrition. However, it is possible that the varieties like Evolution and Revelation that do not respond to phosphite treatment, may lack components in phosphite perception and/or signal transmission.

We also tested the impact of phosphite on root growth in soil by X-ray CT imaging. X-ray CT is a non-invasive method that generates a sequence of images through a soil column and allows the root architecture of plants to be visualised in 3D in their natural environment. We find that at 6 d post-application Δ(D6-D0) there was a significant increase in the root volume and the surface area of the root. There was also a significant increase in seminal and nodal root length and lateral root count six days post phosphite treatment. These results indicate a clear impact of phosphite treatment on early root growth that is likely to improve seedling establishment.

Root system architecture (RSA) determines the distribution of root surface area within the soil profile and so the plant’s capacity to capture nutrients and water (Ho et al., 2004; Hochholdinger & Tuberosa, 2009; Coudert et al., 2010; Gewin, 2010; Bhosale et al., 2018; Giri et al., 2018; Meng et al., 2019). These traits therefore have a direct bearing on crop productivity, particularly under conditions of low resource availability (Lynch, 2011; Lynch, 2013; Lynch et al., 2014; Lynch, 2015). Our nutrition use efficiency experiment further supports this view where we show that phosphite treated plants show a significant increase in root biomass compared to untreated plants when grown under reduced nutrient strength solutions and phosphite treated plants grown in 75% strength nutrient solution outperformed the full strength untreated plants (**Figure 3**).

Improved root traits may also have consequences for synthesis of photosynthesis components and water for leaf gas exchange and thus can explain an increase in photosynthetic capacity, transpiration rate, stomatal conductance and intrinsic leaf water use efficiency WUE of phosphite treated plants (**Figure 4**).

Effects of enhanced photosynthesis can be difficult to interpret due to interactions between enhancement of development in terms of plant size and increased photosynthesis per unit leaf area. We hypothesize that enhanced root morphology can provide greater opportunity to extract water for transpiration, lowering leaf resistance to CO_2_ diffusion and to take up more nutrients to construct greater amounts of photosynthetic components per unit leaf area. Under conditions where resources are restricted these effects could amplify and we see some evidence of this. The difference shown in **Figure 3A** (moderate nutrient depletion) between control and Phi treated plants could be attributed to an increased ability to extract nutrients from the soil. However, the results we saw are not consistent: we would also expect the 25% treatment to also show a difference in *A*_max_. Similarly, we would anticipate an improvement in iWUE would be expected under all treatments.

Our heat stress experiments showed improvements in *Fv/Fm* (an indicator of photoinactivated photosystem II (PSII)) and *V*_cmax_ (the maximum carboxylation rate of the enzyme Rubisco at key points following the heat treatment in phosphite treated plants. These are important findings because both of these processes are heat sensitive and represent a loss of activity in these two key photosynthetic components, PSII and Rubisco. An increased transpiration capacity that could form as a result of an enhanced root system might provide extra leaf cooling and reduce the impact of higher leaf temperatures in the initial phase of the heat treatment. To support this, we observed a significantly higher Gs in the phosphite treated plants compared to untreated at the first time point after the heat was applied. In addition, SIPI measurments revealed reduced senescence in phosphite treated plants (**Figure 5E**). Chlorophyll degradation can occur in cereals under high temperature stress and it is likely that phosphite treatment inhibits high temperature mediated chlorophyll degradation. However, we also cannot rule out an as yet unknown effect of phosphite on stress signalling pathways in the plant.

**Figure 7:**
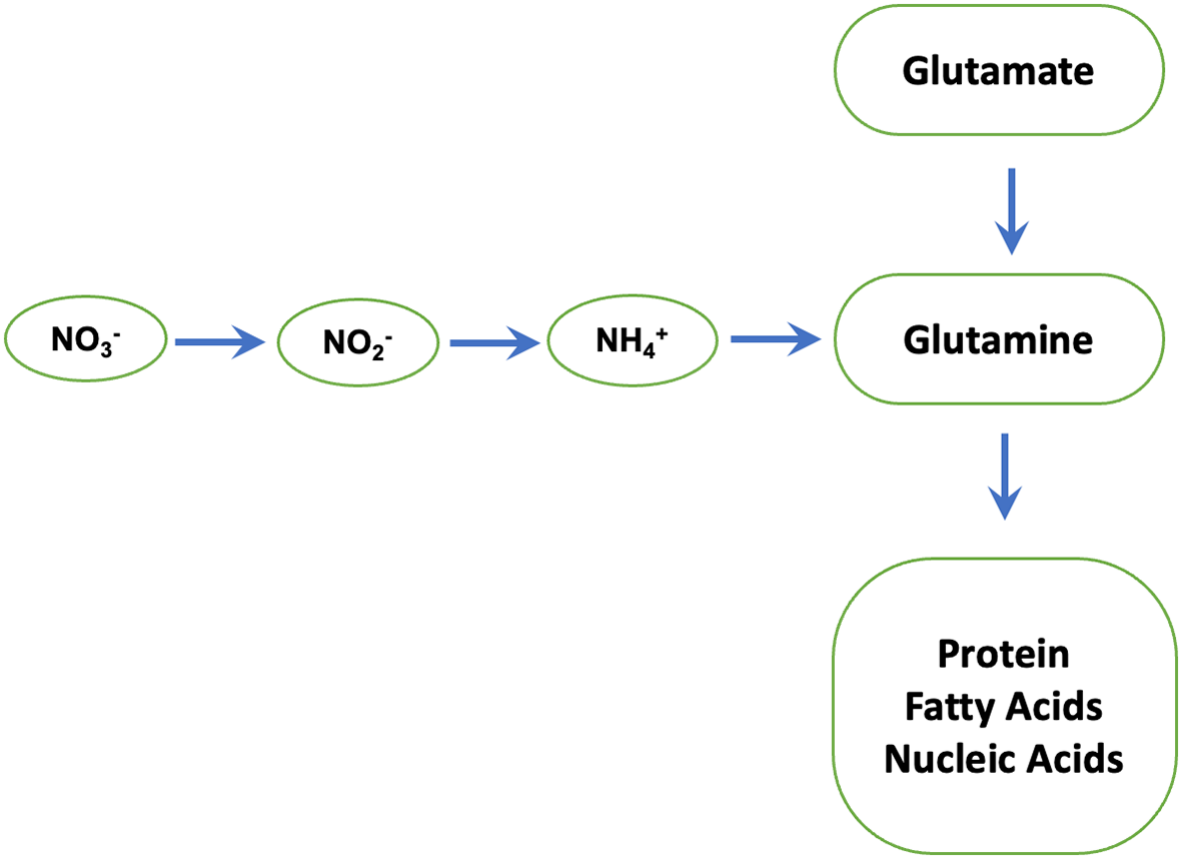
Nitrate reductase: a key enzyme in N metabolism. Nitrate reductase catalyses nitrate to nitrite conversion. The nitrite formed is reduced by the enzyme nitrite reductase to ammonium, which then reacts with glutamate to form glutamine by glutamine synthetase. The latter serves as the amino group donor for the synthesis of amino acids, proteins and Nucleic acids.

We find that phosphites regulate the activity of Nitrate reductase which is a key enzyme in N metabolism and catalyses nitrate to nitrite conversion (**Figure 7**) (Campbell, 1996). The nitrite formed is reduced by the enzyme nitrite reductase to ammonium, which then reacts with glutamate to form glutamine. The latter serves as the amino group donor for the synthesis of amino acids. The total nitrogen flux from nitrate to the amino acids is limited by the activity of the first enzyme nitrate reductase. In plants, the enzyme nitrate reductase therefore has an essential role in providing reduced, metabolizable nitrogen for growth and development and a decisive influence on the increased availability of nitrogen compounds in the plant. Accordingly, an increased nitrate reductase activity leads to increased assimilation of inorganic N to build up plant organs (root, stalk/stem, leaf, and grain/seed) and activity levels have been used as an indicator of plant N content.

Though currently it is not clear how phosphites regulate Nitrate reductase activity. There is some suggestion in the literature between possible link between ion homeostasis and Nitrogen metabolism (Zhao et al., 2019). It was shown that two of wheat Nicotianamine Synthase genes *TaNAS1* and *TaNAS2* are up regulated by increased N application (Zhao et al., 2019). Nicotianamine Synthases are key genes involved in the synthesis of non-proteinogenic amino acid Nicotianamine (Nozoye, 2018; Li et al., 2020). Nicotianamine is known to chelate many metal ions including Iron, Zinc, Copper and Manganese and is involved in internal metal transport (Nozoye, 2018). Crucially, Nicotianamine is involved in acquisition, transport and homeostasis (van Wiren et al., 1999; Pich et al., 2001) of iron, which is required for many of the enzymes involved in Nitrogen assimilation including Nitrate- and Nitrite-reductases (Wang et al., 2003). It has been reported that rice *NAS3* knock outs have reduced shoot and root growth further supporting a link between Nicotianamine Synthases and growth (Aung et al., 2019). In addition, over expression of *NAS* genes has been shown to increase Nicotianamine Synthase levels in several crop plants and confers tolerance to iron deficiency stress (Aung et al., 2019; Nozoye, 2018). Thus, it is possible that phosphite treatment increases Nitrate reductase activity through improved iron homeostasis.

## Conclusions

Our results show that phosphite promotes root growth and improves nutrition use efficiency (root biomass per unit nutrient supply). We also present evidence for improved gas exchange capabilities under conditions of nutrient and water limitation and for enhanced abiotic stress tolerance.

**Figure 8:**
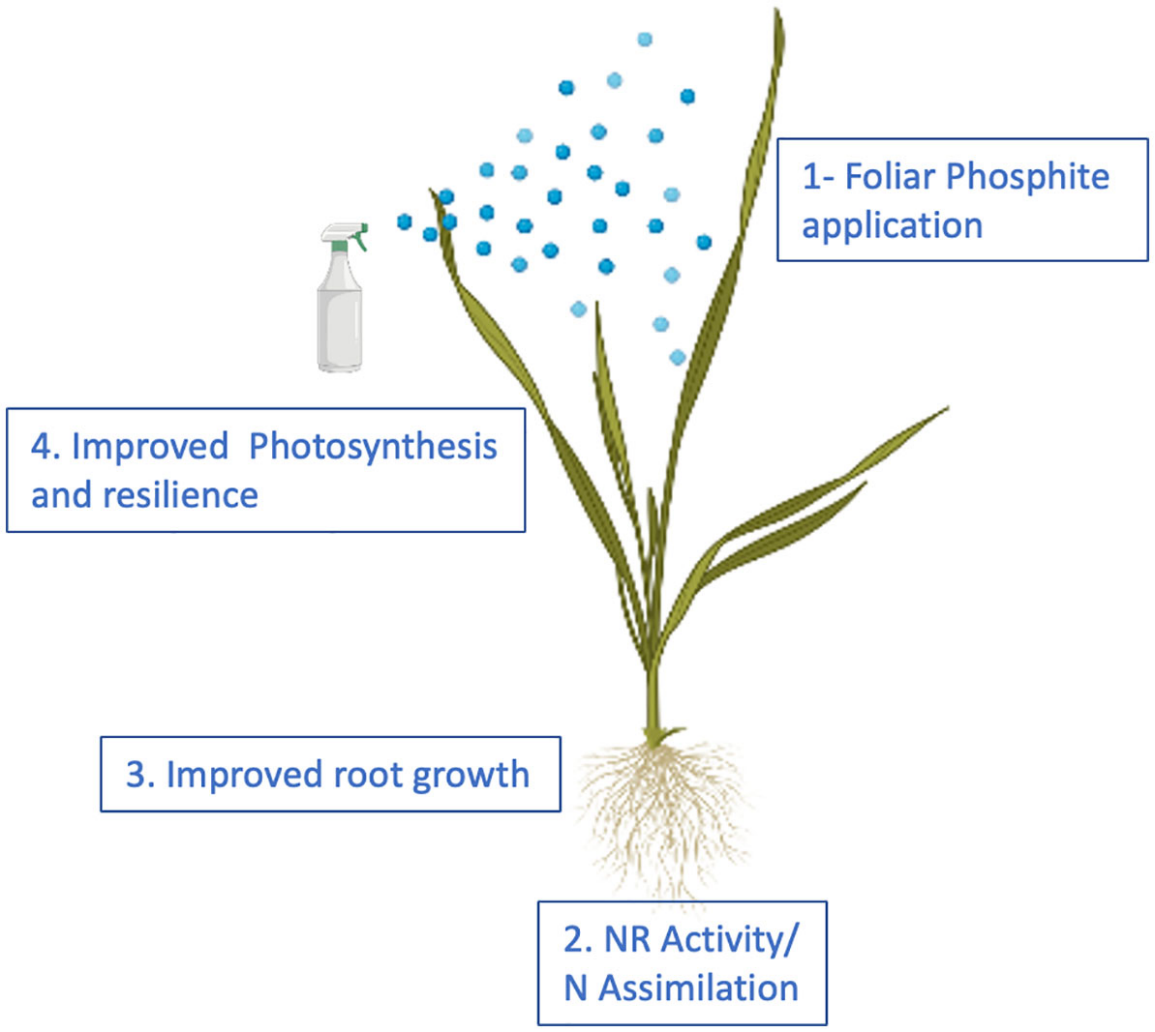
Proposed model for phosphite action. The model provides a temporal framework for impact of phosphite on root growth. Phosphite treatment results in the induction of NAS gene expression and Fe homeostasis to regulate NR activity and root growth.

We propose that phosphite treatment improves plant growth and the ability to survive abiotic stresses through improved Nitrogen and Carbon assimilation thus facilitating improved root growth that in turn improves root biomass, nutrition and water use efficiency (**Figure 8**).

In recent years, there has been a focus towards improving root architecture to enhance crop resource use efficiency. World food production has to increase to meet the demands of growing population and further understanding of the phosphite mediated growth promotion could provide a promising addition to agriculture management practices.

## Data availability statement

The original contributions presented in the study are included in the article/Supplementary Material, further inquiries can be directed to the corresponding author/s.

## Author contributions

UM, JD, SR, KS, NC, RB and RS performed experiments and contributed experimental data. UM, JD, JF, EM and RS designed experiments and analysed data. UM, JD, JF, EM, RB and RS wrote the manuscript. All authors contributed to the article and approved the submitted version.

## Acknowledgments

This work was supported by the awards from the Biotechnology and Biological Sciences Research Council [grant number BB/P010520/1]. This work was also supported by funds from Industrial Partners- Biolchim S.p.A.; Brian Lewis AgricultureT/a Intracrop; Headland Amenity; OMEX Agriculture Ltd; Trade Corporation International SAU; Verdesian Life Sciences Europe Ltd. RB thanks Future Food Beacon Nottingham Research and BBSRC Discovery Fellowship (BB/S011102/1). We also thank Xin for helping in one of the experiments.

## Conflict of interest

The authors declare that the research was conducted in the absence of any commercial or financial relationships that could be constructed as a potential conflict of interest.

This study received funding from Biolchim S.p.A., Brian Lewis Agriculture/Intracrop, Headland Amenity, OMEX Agriculture Ltd, Trade Corporation International SAU and Verdesian Life Sciences Europe Ltd. Except OMEX Agriculture Ltd, no other funders were involved in the study design, collection, analysis, interpretation of data, the writing of this article or the decision to submit it for publication. OMEX Agriculture Ltd. had only the following involvement with the study: They provided plots for field trials, but they were not involved in sample collection, enzyme assays and data analysis presented in **Figure 6B** and this was all done by Nottingham.

## Publisher’s note

All claims expressed in this article are solely those of the authors and do not necessarily represent those of their affiliated organizations, or those of the publisher, the editors and the reviewers. Any product that may be evaluated in this article, or claim that may be made by its manufacturer, is not guaranteed or endorsed by the publisher.
