# Supplementary material for: Phosphite treatment can improve root biomass and nutrition use efficiency in wheat
Source: Front Plant Sci. 2022 Oct 31;13:1017048. doi: 10.3389/fpls.2022.1017048 (PMC9662169; doi:10.3389/fpls.2022.1017048)
Supplement: Supplementary file 1 [file Presentation_1.pptx]

## Slide 1
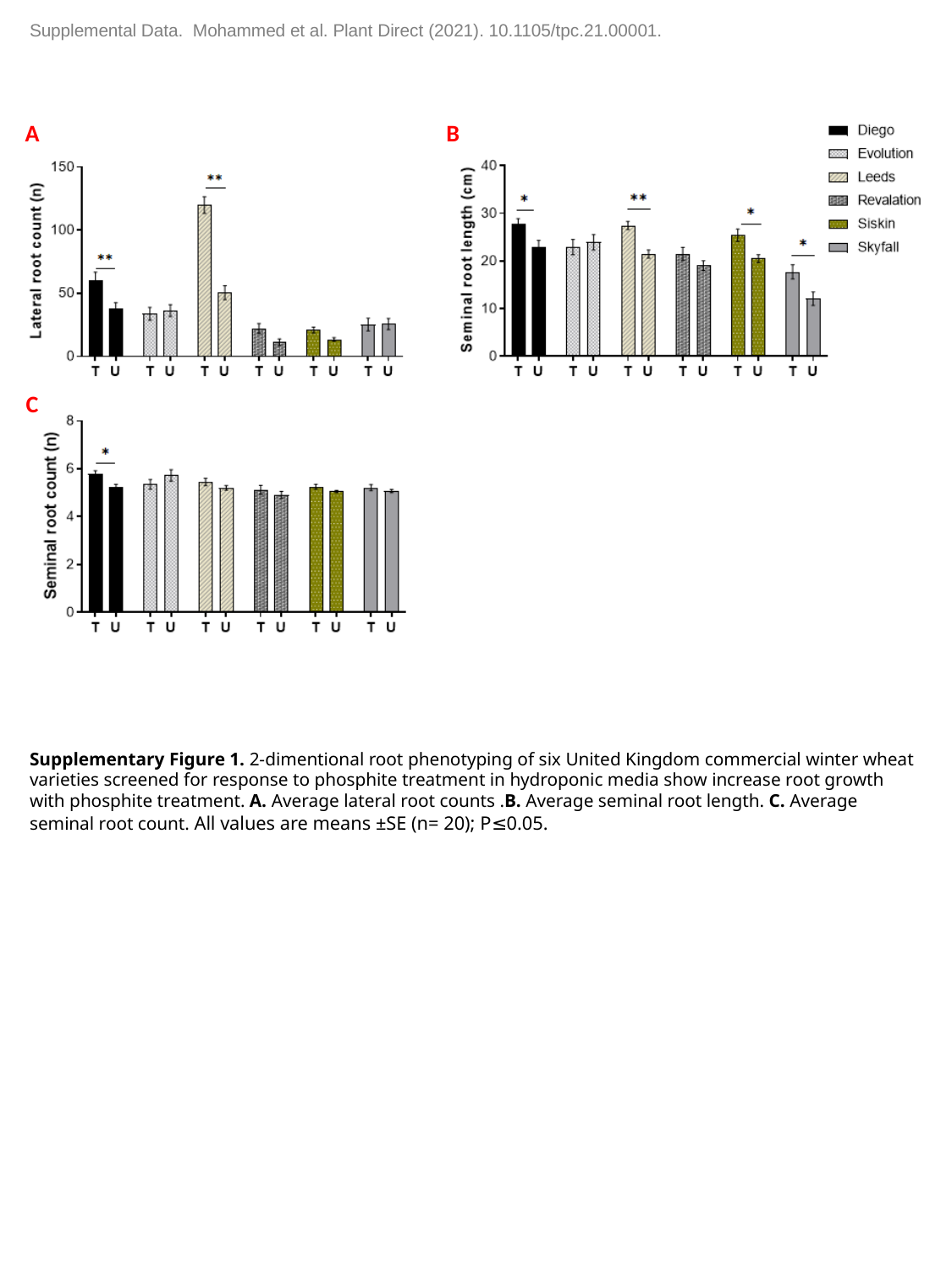

Supplemental Data. Mohammed et al. Plant Direct (2021). 10.1105/tpc.21.00001.
A
B
C
Supplementary Figure 1. 2-dimentional root phenotyping of six United Kingdom commercial winter wheat varieties screened for response to phosphite treatment in hydroponic media show increase root growth with phosphite treatment. A. Average lateral root counts .B. Average seminal root length. C. Average seminal root count. All values are means ±SE (n= 20); P≤0.05.

## Slide 2
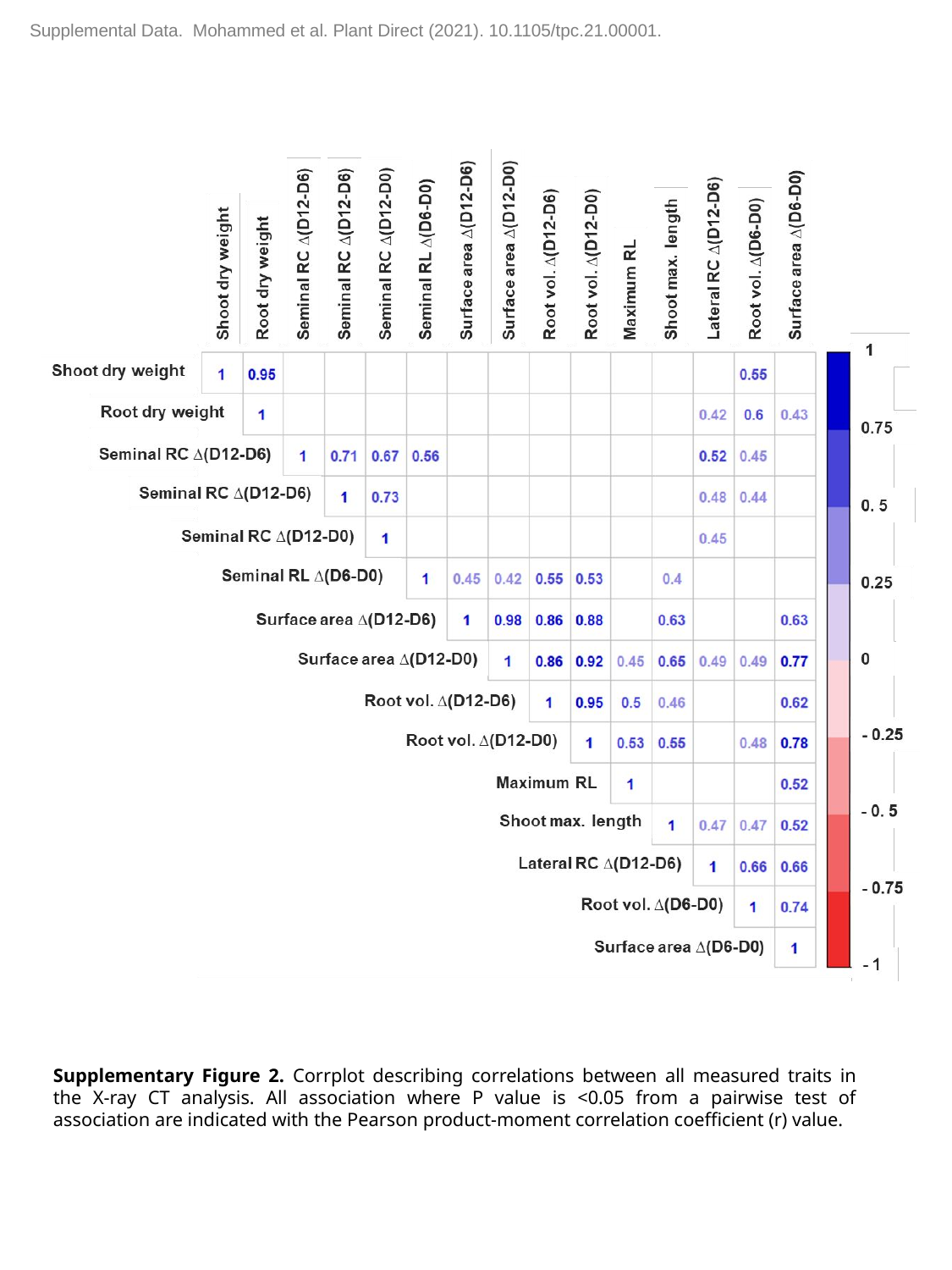

Supplemental Data. Mohammed et al. Plant Direct (2021). 10.1105/tpc.21.00001.
Supplementary Figure 2. Corrplot describing correlations between all measured traits in the X-ray CT analysis. All association where P value is <0.05 from a pairwise test of association are indicated with the Pearson product-moment correlation coefficient (r) value.

## Slide 3
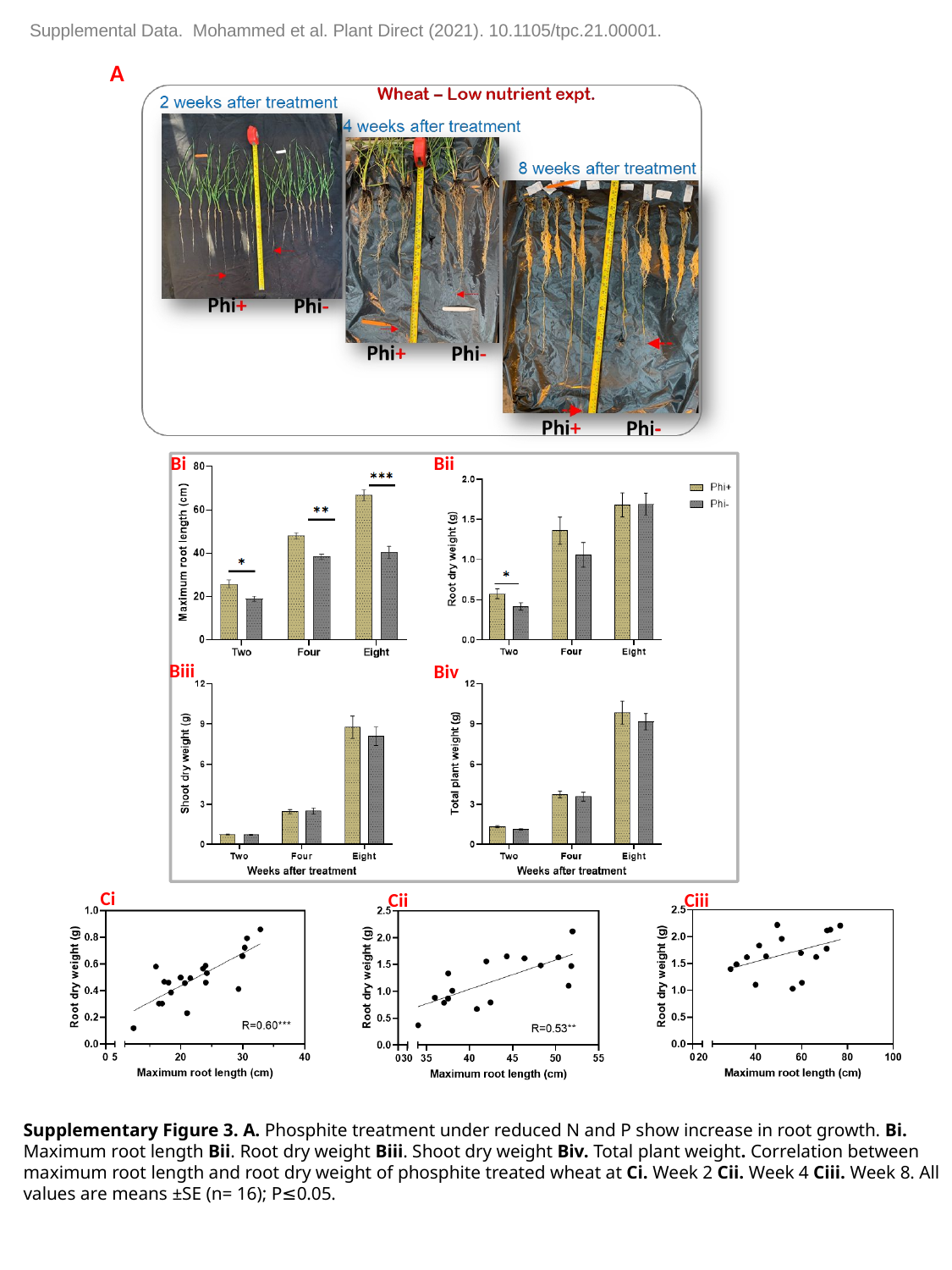

Supplemental Data. Mohammed et al. Plant Direct (2021). 10.1105/tpc.21.00001.
A
Bi
Bii
Biii
Biv
Ci
Cii
Ciii
Supplementary Figure 3. A. Phosphite treatment under reduced N and P show increase in root growth. Bi. Maximum root length Bii. Root dry weight Biii. Shoot dry weight Biv. Total plant weight. Correlation between maximum root length and root dry weight of phosphite treated wheat at Ci. Week 2 Cii. Week 4 Ciii. Week 8. All values are means ±SE (n= 16); P≤0.05.

## Slide 4
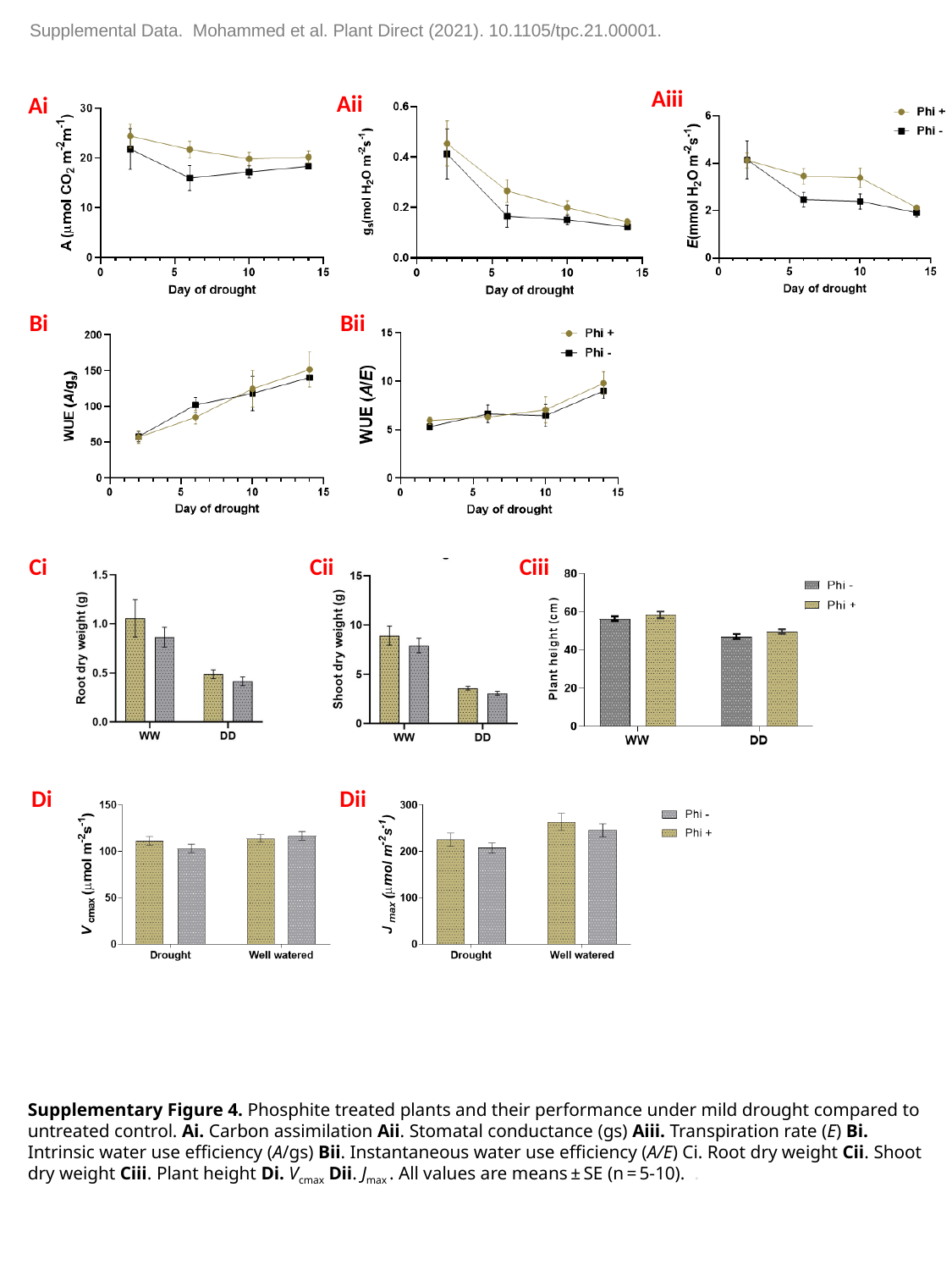

Supplemental Data. Mohammed et al. Plant Direct (2021). 10.1105/tpc.21.00001.
Aiii
Aii
Ai
Bi
Bii
Ci
Cii
Ciii
Di
Dii
Supplementary Figure 4. Phosphite treated plants and their performance under mild drought compared to untreated control. Ai. Carbon assimilation Aii. Stomatal conductance (gs) Aiii. Transpiration rate (E) Bi. Intrinsic water use efficiency (A/gs) Bii. Instantaneous water use efficiency (A/E) Ci. Root dry weight Cii. Shoot dry weight Ciii. Plant height Di. Vcmax Dii. Jmax . All values are means ± SE (n = 5-10). .

## Slide 5
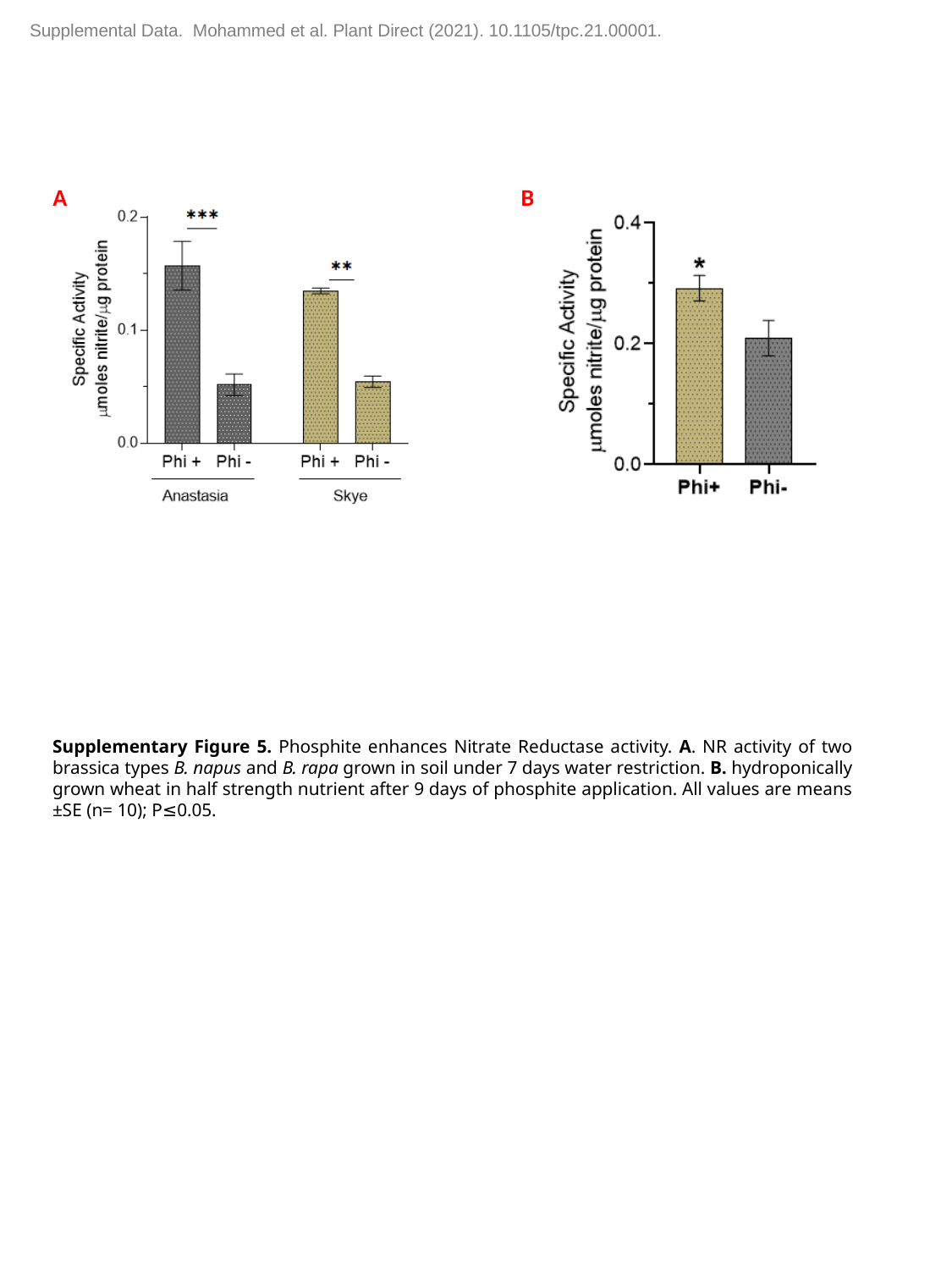

Supplemental Data. Mohammed et al. Plant Direct (2021). 10.1105/tpc.21.00001.
A
B
Supplementary Figure 5. Phosphite enhances Nitrate Reductase activity. A. NR activity of two brassica types B. napus and B. rapa grown in soil under 7 days water restriction. B. hydroponically grown wheat in half strength nutrient after 9 days of phosphite application. All values are means ±SE (n= 10); P≤0.05.
